# The future backbone of nutritional science: integrating public health priorities with system-oriented precision nutrition

**DOI:** 10.1017/S0007114524001466

**Published:** 2024-09-14

**Authors:** Guy Vergères, Murielle Bochud, Corinne Jotterand Chaparro, Diego Moretti, Giulia Pestoni, Nicole Probst-Hensch, Serge Rezzi, Sabine Rohrmann, Wolfram M. Brück

**Affiliations:** 1 Agroscope, Bern, Switzerland; 2 Unisanté, University Center for Primary Care and Public Health, University of Lausanne, Lausanne, Switzerland; 3 Department of Nutrition and Dietetics, Geneva School of Health Sciences, HES-SO University of Applied Sciences and Arts Western Switzerland, Geneva, Switzerland; 4 Nutrition Group, Swiss Distance University of Applied Sciences (FFHS)/University of Applied Sciences and Arts of Southern Switzerland (SUPSI), Zurich, Switzerland; 5 Swiss Tropical and Public Health Institute, Allschwil, Switzerland; 6 University of Basel, Basel, Switzerland; 7 Swiss Nutrition and Health Foundation, Epalinges, Switzerland; 8 Epidemiology, Biostatistics and Prevention Institute (EBPI), University of Zurich, Zürich, Switzerland; 9 Institute for Life Sciences, University of Applied Sciences Western Switzerland Valais-Wallis, Sion, Switzerland

**Keywords:** Nutrition, Epidemiology, Digitalisation, Health, Switzerland

## Abstract

Adopting policies that promote health for the entire biosphere (One Health) requires human societies to transition towards a more sustainable food supply as well as to deepen the understanding of the metabolic and health effects of evolving food habits. At the same time, life sciences are experiencing rapid and groundbreaking technological developments, in particular in laboratory analytics and biocomputing, placing nutrition research in an unprecedented position to produce knowledge that can be translated into practice in line with One Health policies. In this dynamic context, nutrition research needs to be strategically organised to respond to these societal expectations. One key element of this strategy is to integrate precision nutrition into epidemiological research. This position article therefore reviews the recent developments in nutrition research and proposes how they could be integrated into cohort studies, with a focus on the Swiss research landscape specifically.

## Importance of nutrition to human health

In 1912, the term ‘vitamine’ was coined leading to the discovery of the first vitamin (vitamin B_1_ also called thiamine) in 1926^(1)^. Modern nutrition science, thus, first focused on the discovery, description and treatment of diseases and conditions due to single nutrient deficiencies, the definition of recommended daily allowances and food fortification. Over the decades, the focus changed, and nutrition science confronted the challenge of studying the associations of foods and dietary patterns with cardiometabolic diseases and other chronic diseases, using data from prospective population-based cohorts or intervention studies^(2)^.

The Global Burden of Disease study provides the most comprehensive estimates of diet-related burden worldwide. It is clear from the Global Burden of Disease study that there is enormous potential to improve population health by reducing nutritional risks and seizing nutritional opportunities. According to the Institute for Health Metrics and Evaluation, diet was the third highest risk for the Global Burden of Disease in 2019, following high blood pressure and tobacco. In 2017, 22 % of all deaths were attributable to an unbalanced diet, mainly via the increased risk of CVD^(3)^. Overall, it is estimated that obesity was responsible for 160 million disability-adjusted life years (DALY) and 5 million deaths in 2019, with a high burden across all regions of the world^(4)^. Obesity-related DALY and mortality are expected to increase by nearly 40 % in the coming decade^(4)^.

Nutrition is on the top priority list of many supranational organisations such as the WHO, which defined objectives to promote healthy nutrition and decrease the risk of noncommunicable diseases that should take place within the boundaries of sustainable development^(5)^. The 2030 Agenda for Sustainable Development, adopted by all United Nations Member States in 2015, includes several goals related to nutrition such as ‘End hunger, achieve food security and improved nutrition and promote sustainable agriculture’ or ‘Ensure healthy lives and promote well-being for all at all ages’ (United Nations Decade of Action on Nutrition 2016–2025: https://www.un.org/nutrition/; WHO 2030 Agenda for Sustainable Development: https://www.who.int/europe/about-us/our-work/sustainable-development-goals). One-third of all man-made greenhouse gas emissions are a result of food systems^(6,7)^. The EAT Lancet Commission report stated that about half of man-made greenhouse gas emissions could be attributable to food choices by 2050^(8)^. Considering the climate crisis, diets are likely to substantially change in the coming years. This emphasises the importance to continuously monitor what people eat and to continuously assess links between diet and health status.

In Switzerland today, food is always available in great variety and abundance, but many people still consume an unbalanced diet including excessive intake of salt, sugar and fatty foods, which increase the risks of developing noncommunicable diseases such as diabetes, obesity or CVD. In addition to the human suffering they cause, such diseases account for around 80 % of Swiss healthcare costs. While data on nutritional deficiency are lacking on a country level, it has been shown in smaller studies that vitamin D, folic acid and Fe are at-risk nutrients also in the Swiss population, in particularly in more vulnerable populations sub-groups^(9–11)^. Also, iodine, a historical public health focus in Switzerland and a global success story, requires constant monitoring^(12)^. To prevent nutrition-related diseases, the Swiss Nutrition Policy 2017–2024 has been developed along with an action plan focusing on four actions areas, that is, information and education; framework conditions; coordination and cooperation and monitoring and research. In addition, a strategy and action plan have been developed to promote the implementation of the UN 2030 Agenda for Sustainable Development. In the field of research, it is important to have a coherent contribution of the key actors in nutrition, especially researchers from main institutions in Switzerland. To reach this goal, an initiative has been developed, the Swiss Research Network-Healthy Nutrition^(13)^.

## Current status of epidemiological nutrition research in Switzerland

To date, various population-based studies collecting information on diet or nutritional status have been conducted in Switzerland. Table 1 summarises studies with assessment of diet, whereas Table 2 provides an overview of studies with assessment of specific food items intake or nutrient status conducted in Switzerland in the last decades.

**Table 1.** Overview of population-based studies conducted in Switzerland including assessment of the diet of participants

Ref.	Study	Study design	Region	Year	Sample size (∼n)	Age (years)	Dietary assessment	Biological sample
^(14,15)^	Monitoring of Trends and Determinants in Cardiovascular Disease (MONICA)	Three surveys with mortality follow-up via linkage with the Swiss National Cohort	Canton Vaud/Fribourg, Canton Ticino	1984–1993	10’000	25–74	Simplified 24-h recall checklist with ‘yes’ or ‘no’ questions about the intake of specific foods	Blood
^(16)^	National Research Program 1A (NRP1A)	Intervention study with mortality follow-up via linkage	Two towns in the French- and two in the German-speaking Switzerland	1977–1980	8’600	16–92	Simplified 24-h recall checklist with ‘yes’ or ‘no’ questions about the intake of specific foods	Blood
^(17)^	Swiss Health Survey (SHS)	Cross-sectional, repeated	Switzerland	1992-ongoing (every 5 years)	22’000 (in 2022)	> 15	Short questions about consumption of broadly defined food groups, changing across waves, including the frequency and occasionally the portion size consumed	
^(18)^	Swiss Food Panel 1·0	Longitudinal study	German- and French-speaking Switzerland	2010–2011	6100	> 20	Short FFQ, including foods known to have unfavourable health effects or food groups established by dietary guidelines, and assessing portion sizes only for fruit and vegetables	
^(19)^	Swiss Food Panel 2·0	Longitudinal study	German- and French-speaking Switzerland	2017–2019	5’500	> 20	Semi-quantitative FFQ	
^(20,21)^	Bus Santé	Cross-sectional, repeated	Geneva	1992-ongoing	>20’000	35–74	Semi-quantitative FFQ	Capillary blood
^(22–24)^	CoLaus	Longitudinal study	Lausanne	2003-ongoing	6’700	35–75	Semi-quantitative FFQ	Blood, urine, saliva, genetic data
	PsyCoLaus				5’100	35–66		
^(25,26)^	Sapaldia 3	Longitudinal study	Switzerland	2010-ongoing	6’000	38–80	Semi-quantitative FFQ	Blood
^(27)^	National Nutrition Survey menuCH	Cross-sectional	Switzerland	2014–2015	2’000	18–75	24-h dietary recalls	
^(28)^	menuCH Kids	Cross-sectional	Switzerland	2023–2025	1’800	6–17	24-h dietary recalls	Blood, urine, on a voluntary basis

NRP1A MONItoring of trends and determinants in CArdiovascular disease.

**Table 2.** Overview of population-based studies conducted in Switzerland including the assessment of specific food items intake or nutrients status

Ref.	Study	Study design	Region	Year	Sample size (∼n)	Age (years)	Dietary assessment	Biological sample
^(29)^	Survey on dietary supplements	Cross-sectional	Switzerland	2022	1’200	18–75	Questionnaires	
^(30–32))^	Study on salt consumption	Cross-sectional	Switzerland	2010–2011	1’400	> 15	Questionnaire on dietary habits	Urine
				2021–2022				
^(33,34))^	Se biomonitoring	Cross-sectional	Switzerland	1992–1993	600	20–40		Blood
				2005–2006	800	18–72		
				2021–2022	700			
^(35)^	Iodine biomonitoring (in children and pregnant women)	Cross-sectional	Switzerland	2020–2022	700	6–12	Questionnaire on consumption of iodine-rich foods	Urine, dry blood samples in pregnant women
				Every 5 years	500	18–44		
^(36)^	Nutriserocovid (Zn, Se, Cu and vitamin D)	Cross-sectional	Canton of Vaud	2020	932	>18	No questionnaire on diet	Blood

The MONItoring of trends and determinants in CArdiovascular disease^(14)^ and the National Research Project 1A^(16)^ were two population-based studies aiming to investigate cardiovascular and lifestyle risk factors in Switzerland between the 1970s and 1990s. In both studies, a mortality follow-up was established through linkage of census and mortality data, and simplified food checklists with yes/no questions on the consumption of specific foods were used to assess diet^(14–16)^. The Swiss Health Survey is a nationally representative cross-sectional survey, conducted every 5 years since 1992, with the aim to collect information on the health status of the Swiss population^(17)^. The dietary assessment is conducted as part of an extensive questionnaire on health behaviours, via short questions on the consumption of selected food groups. The Swiss Food Panel 1·0 and 2·0 are two longitudinal studies focusing on eating behaviours and covering the German- and French-speaking part of the country^(18,19)^. The dietary assessment methods used differed slightly among the two studies: the Swiss Food Panel 1·0 used a FFQ specifically developed for the study and considering food groups with unfavourable health effects or established by dietary guidelines, whereas the Swiss Food Panel 2·0 used a semi-quantitative FFQ adapted from the US Nurses’ Health Study and inquiring about the consumption of forty-seven types of food and beverages^(18,19,37,38)^.

Four studies used a validated semi-quantitative FFQ to assess diet. The Bus Santé study is a community-based long-term survey, designed to assess cardiovascular risk factors of the population of Geneva,^(20)^ CoLaus^(22)^ and PsyCoLaus^(23)^ represent two components of a single-centre cohort aiming at assessing risk factors for CVD and psychiatric disorders, respectively, in the population of Lausanne aged 35–75 years at baseline^(39)^. Sapaldia 3 is the third follow-up of the nationally representative Sapaldia cohort study with associated biobank designed to explore broad health and ageing effects of air pollution and the exposome more broadly^(25,40)^.

Finally, the first National Nutrition Survey of the Swiss adult population was conducted in 2014/2015 (menuCH)^(27)^, and an analogous study in children is conducted in 2023/2024 (menuCH-Kids)^(28)^. Both studies are cross-sectional and assess diet using two non-consecutive 24-h dietary recalls, providing crucial insights into the food consumption and dietary habits of the Swiss adult and children populations.

With respect to the assessment of specific food items or nutrients, only Se, iodine and Zn status have been measured in the Swiss population on a national level^(33,35)^. Additionally, a survey on the intake of dietary supplements was recently conducted by the Federal Food Safety and Veterinary Office^(29)^, and a population-based survey investigating the consumption of salt in the Swiss population was conducted twice^(30)^.

## Gaps in Swiss nutrition research

### Large-scale Swiss longitudinal data

Only few studies described above provide comprehensive dietary data collected using validated dietary assessment methods and were conducted nationally and longitudinally. As shown in Tables 1 and 2, Switzerland has conducted several studies that assessed diet. They do, however, differ strongly with respect to how diet was assessed and, thus, the quality of dietary information. A major disadvantage of most of these studies is that they were not intended to be cohort studies (with the exceptions of SAPALDIA, CoLaus-PsyCoLaus, Swiss Food Panels). Existing cohorts only cover parts of the Swiss population and are limited in size. However, Switzerland has three main language regions with cultural habits mirroring those of the large neighbouring countries, which influences the dietary habits of the population^(41)^. It is therefore important to conduct geographically comprehensive health and nutrition studies.

Some of these studies used a rather crude dietary assessment method, for example, checklists with ‘yes’ or ‘no’ questions on the intake of specific foods^(14,16)^, short questions on the consumption frequency and occasionally quantity of broadly defined food groups^(42)^, or focusing on specific foods only^(18)^.

Although menuCH^(27)^ and menuCH-Kids^(28)^ provide essential detailed and nationally representative data on the food consumption and dietary habits of the adult and children population of Switzerland, they are cross-sectional^(27,28)^ and, in the case of menuCH, do not include the collection of biological samples^(27)^.

Most studies that have assessed dietary information in Switzerland were mainly conducted among adults. menuCH Kids^(28)^ will at least partly fill this gap. However, studies with a life-course approach are missing in Switzerland. Nutritional trends such as an increasing prevalence of plant-based diets (vegans, vegetarians, flexitarians), the consumption of plant alternatives for meat and dairy, but also cooking skills and nutrition literacy are likely to differ between age groups and generations^(43,44)^. This underlines the importance for cohorts that do not only include middle-aged populations that are vulnerable for chronic diseases in the near future, but also younger and older populations. Besides missing information on some age groups, studies in pregnant and lactating women, people with handicaps, or people with migration backgrounds are missing.

Misreporting of nutritional intake is a major limitation of current nutritional studies. For example, Subar and colleagues^(45)^ reported that energy intake is considerably underreported on 24h recalls (12–14 % for men; 16–20 % for women) and even more on FFQ (31–36 % for men, 34–38 % for women). The direct assessment valid biomarkers of nutrients and food intake^(45,46)^ (see sections Biomarkers and Reference methods) are thus key to more objectively measure dietary intake. In the future, the possibility of incorporating machine-learning based methods aimed reducing various sources of misreporting^(47)^ is worthy of investigation.

In addition to the above gaps, Swiss studies that assess dietary information hardly ever allow linking dietary data with health outcomes. For example, menuCH cannot be linked to health outcomes on an individual level. Even if this limitation was overcome by using geographical linking methods^(48,49)^, individual level data would allow for increased precision and the possibility to better adjust for potential confounders. Also, although the cohorts Colaus-PsyColaus and Sapaldia have been linked to health outcomes, they are restricted to specific regions and only few clinical conditions can be studied. Larger surveys, including the Swiss Health Surveys, can be linked to mortality and cancer incidence using established linking methods, but are limited by crude dietary assessments^(50–53)^.

The existing Swiss cohorts have the potential to provide important information in the future. In particular, several of these cohorts already integrate repeated measures in their design (see Table 1); resurvey of existing study participants could help to address many issues around changing diets across differences ages or time periods. These possibilities are however limited as none of the studies above is currently able to address questions of nutritional transition with respect to, for example, (i) the health impacts of poorly (ultra) processed foods, (ii) the persistence of nutritionally caused cardiometabolic conditions and (iii) climate change and the insurgence of plant-based diets and the potential lower nutritional value of plant-based meat and milk alternatives, and (iv) the overall consequences and extent of a broader uptake of vegan and vegetarian diets.

### Quality of the Swiss food composition database

High-quality food composition databases are crucial in nutrition research to ensure accurate and reliable results, regardless of the dietary assessment methods used to record food consumption^(54)^. Food composition databases should be updated frequently to ensure a good coverage of new products entering the market. In addition, they should ideally include a variety of different foods and a wide range of nutrients, vitamins, and minerals, as well as other food components linked to health outcomes. The Swiss Food Composition Database (https://naehrwertdaten.ch/en/) currently includes around 1’100 different generic foods and provides data for 40 nutrients. These numbers are rather low when compared to food composition databases from the large neighbouring countries Germany (approximately 15’000 foods and approximately 140 nutrients), France (approximately 3’200 foods and approximately 70 nutrients) and Italy (approximately 1’000 foods and approximately 120 nutrients). Compared to these food composition databases, the Swiss database lacks specific data for fatty acids (e.g. *n*-3, *n*-6, EPA and DHA), sugars (e.g. fructose, lactose and glucose), proteins (e.g. amino acids), trace elements (e.g. manganese and copper), and vitamins (e.g. vitamin K, including vitamin K_2_ and vitamin B_7_). Furthermore, anti-nutritive factors such as polyphenols, lectins, saponins or phytic acid, which are essential to calculate the nutrient bioaccessibility and bioavailability of key nutrients are not present in the database. The Swiss Food Composition Database should therefore be further developed and regularly updated, and resources invested in its maintenance.

### Understanding consumer choices

The Swiss Food Panels by the ETH Zürich, among others, aims at connecting dietary habits and food consumption with aspects of food literacy^(37)^, drivers of food consumption^(55)^, and predictors of diet quality^(56)^. Besides the Food Panels, no studies targeted psychological aspects of diet and food consumption to address questions such as ‘why do consumers choose foods’, ‘what drives eating patterns’ etc.

Even though Switzerland is a country with high mean household income and general mandatory health insurance, there are disparities in health and access to health care^(57–59)^. Dietary habits are known to differ by region and by socioeconomic status, but what is missing is whether these differences are due to lack of knowledge (‘nutrition literacy’) or differences in accessibility of food (‘food deserts’) or lack of infrastructure in rural areas, or a mix of these factors. There is also no data available on overall cooking skills and ability as well as barriers to implement healthy nutrition in a private household. It will thus be important to conduct longitudinal research studies on dietary habits to understand how Swiss consumers actually handle food. This becomes even more important with respect to challenges due to climate change and the need for a transformation of the worldwide food system^(8)^.

### Filling the gap with a Swiss nutrition cohort

State-of-the art assessments of diet and food environments are crucial in any large-scale population-based cohort like that one that is foreseen in Switzerland^(60)^. A regular assessment of diet will help to overcome the limitations of existing studies as mentioned above. We argue that an investment in setting up a nutrition cohort in Switzerland is crucial. Ideally, such a cohort is implemented into the planned large-scale population-based Swiss Cohort & Biobank.

The model proposed in the Swiss Cohort & Biobank White Paper^(60)^ is one of an internationally harmonised, large-scale (i.e. over 100’000 participants) long-term prospective population-based cohort covering all age groups. Such a cohort would provide the necessary data to support evidence-based policies, conduct population-based surveillance, and advance public health knowledge within the Swiss context. The large cohort is complemented by selected sub-cohorts targeting specific populations of interest (e.g. pregnant women, patients, vegetarians/vegans, etc). Such a cohort would allow for covering topics related to prevention, including risk and disease screening, and health promotion aiming at producing the evidence to implement health-in-all policies in Switzerland. The planned collection of medical imaging and biological samples would allow for producing population-based reference data, including in the field of nutrition. We here present and discuss in detail some of the methods that we consider most relevant for such a national project.

## Laboratory tools for a nutritional cohort

### Dietary intake assessment tools

Dietary intakes vary daily, across seasons and ages. Regional eating habits, increasing heterogeneity as in Switzerland, may further complicate the measurement process. In addition, individuals consume multiple foods and beverages with varying nutrient profiles and may consume dietary supplements.

To collect dietary intake data, researchers use self-report tools, which are affected by different types and degrees of measurement error^(61)^. This leads to the suggestion of complementing them with objective measures (see Section Analytical targets of personalised nutrition). Nonetheless, validated biomarkers that reflect true intake are known for only few dietary components and mostly reflect recent food intake, and objective measures alone do not provide insights into what people actually consume and the related contextual factors. Thus, there is a clear value in the continued use of dietary intake self-report tools, acknowledging their strengths and limitations^(62)^.

Established methods of dietary intake assessment in research mainly include FFQ, 24-h dietary recalls, (weighted) food records, and screeners^(63)^. Besides their risk of recall bias, all these methods have specific advantages and drawbacks. For surveys, the European Food Safety Authority recommends to keep the burden for participants at a minimum by using two non-consecutive 24-hour dietary recalls in adults, and the 24-hour dietary recall method followed by a computer-assisted personal or telephone interview in infants and children^(64)^. A short food propensity questionnaire is also recommended to collect information on the consumption of some less frequently eaten foods and food supplements. The combination of both allows for computing a participant’s habitual diet using methods such as the Multiple Source Method^(65)^. Various new technologies have emerged to collect dietary intake, including web-based dietary assessments with self-administered record such as myfood24 or the Automated Self-Administered 24-Hour Dietary Recall^(66,67)^.

In Switzerland, some studies used an electronic FFQ. Automated Self-Administered 24-Hour Dietary Recall is tested in different subgroups of the populations including children, adolescents, adults, and elderly^(68)^ and accuracy of the automated dietary app MyFoodRepo is evaluated against controlled reference values from weighted food diaries^(69)^. A multilingual (German, French) web-based FFQ for adults that captures food consumption of the past four weeks has been developed^(70)^ and its validation is underway.

### Biosampling

Biological sample collection is a critical factor in study design. Various factors such as nutritional status, physical activity, and circadian rhythm, and the exposome more broadly can significantly influence metabolite levels and leave molecular fingerprints in human tissues and fluids^(71)^. However, the process of collection also alters the sample due to necessary or incidental additives in collection containers and sampling devices.

Blood is considered rich in information for clinical chemistry-based research and provides a temporal snapshot of an individual’s physical condition.^(72–74)^. Drawing large volumes of blood should be avoided especially in vulnerable subjects or where venous access is difficult as in children or the critically ill^(72)^. To minimise the blood sample volume, capillary blood sampling and blood spot cards have been introduced for blood collection^(75,76)^. These techniques have been applied in point-of-care assessments, drug development, medical monitoring, and nutritional studies but has been linked to analyte loss^(76,77)^. Because of the relatively large volume of blood required for studies conducting a broad range of analytical tests, for example, multi-omics studies, traditional blood sampling remains the method of choice. However, the sensitivity of analytical technologies constantly improves as illustrated by the development of nanomaterials-assisted proteomics and metabolomics^(78)^ as well as single-cell analysis of omics datasets^(79)^.

Urine has been regarded as a reservoir for numerous metabolites originating from exogenous nutrients and drugs or endogenous substances^(80)^. While spot urine (urine taken at a specified time of the day) collection is common, a 24-hour urine collection (pool of all voids within a 24-hour period), is considered the ‘gold standard’^(81–83)^. However, a complete urine collection in a 24-hour time interval may be difficult to obtain, especially when proper sample storage and transportation are considered to maintain sample integrity^(73,84,85)^.

Saliva offers a less invasive yet powerful alternative to blood for clinical applications^(86,87)^. The amount and composition of proteins in saliva vary according to circadian rhythm, diet, age, sex, and physiology^(88)^. Molecules are generally found in nano- or picograms which makes the reliable detection of biologically active molecules difficult^(89,90)^. The standardisation of saliva collection protocols reduces the high variation of saliva parameters and facilitates downstream analysis^(91)^.

Less commonly investigated human tissues offer interesting complementary sources of biomarkers whose potential should be further investigated. For example, the carbon and nitrogen stable isotope ratios 13C/12C and 15N/14N in hairs can be used as dietary marker^(92)^; nails emerge as an adequate matrix to evaluate the nutritional status of zinc^(93)^; also the nutritional status of carotenoids can be measured by evaluating this compound in skin^(94)^.

A wide range of metabolic products are produced by the microorganisms within the gut and are important to human health^(95)^. While faeces are readily accessible, the recruitment of individuals willing to participate in a study may be difficult due to due to various barriers. For metabolome studies, the most common practice is to freeze samples at –80°C, –40°C, or –20°C, sometimes aided by flash freezing in liquid nitrogen as it is unclear if stabilizing solutions adversely affect metabolite profiles^(96)^.

### Biomarkers

The classical measurement of food and nutritional intake are self-reported food intake measurements^(97)^. While these have inherent limitations, the use of biomarkers enables the objective measurement of nutrient intake. Biomarkers are indicators that can be measured to inform about the normality or the disfunction of specific biological processes in response to multiple environmental and/or genetic factors such as gene polymorphisms, diet (e.g. nutrient intake and levels in the body), physiological status (e.g. pregnancy, lactation, ovarian cycle and menopause, physical exercise), physical and chemical exposures (e.g. environmental pollutants), lifestyle (e.g. stress levels) and various pathogenic processes and diseases. In nutrition, biomarkers can be either direct (e.g. nutrient itself) or indirect (e.g. nutrient-associated endpoints or functional biomarkers) measurements of the nutrient(s) of interest. Nowadays, a series of biomarkers are available in routine for both nutrition clinical practices and research. Such conventional biomarkers that are based on single nutrients show limitations and encourage the developments of a new generation of biomarkers that better reflect the metabolic processes in relation to diets. Metabolomic approaches enable to simultaneously quantify multiple metabolites representative of the systemic metabolic regulatory processes (see section Metabotypes). This makes metabolomics a suitable approach to discover new nutrient-associated metabolic patterns and thus additional functional biomarkers for nutrition. Whatever their direct or functional nature, biomarkers must fulfil the following specifications:

- correlation with the rate of nutrient intake, at least within the nutritionally significant range, and respond to deprivation of the nutrient;
- acceptable specificity and selectivity for the nutrient(s) of interest;
- relation to a meaningful period of time;
- indication of normal physiological function;
- measurable in an accessible biological sample (e.g. typically blood and urine);
- validated analytical method (linearity, accuracy, reproducibility) deployable in routine and at affordable cost;
- availability of established normative data.

#### Classical biomarker measurement

Biomarkers of nutrient status measure the level of biological adequacy of nutrients in the organisms, for example, vitamin status biomarkers. Selected status biomarkers for micronutrients are reported in Table 3. Although classical biomarkers have advantages to be widely deployed in routine analysis for both general population and patient groups^(98)^ it is worth mentioning that basically all biomarkers have limitations and special attention needs to be paid to their interpretation.

**Table 3.** Examples of direct and functional biomarkers of micronutrient status

Micronutrient (vitamin)	Biomarkers*	Micronutrient (mineral)	Biomarkers*
Vitamin A	Serum/plasma retinol Total body store using stable isotopes Change in serum retinol after oral load Liver retinyl esters	Se	Plasma Se Plasma selenoprotein P (functional biomarker)
Vitamin D	Serum total 25-(OH)-vitamin D (sum of D_2_ and D_3_ forms)	Iodine	Urinary iodine
Vitamin E	Serum tocopherols (often a- and g-tocopherol) Erythrocyte haemolysis (functional biomarker)	Zinc	Serum/plasma Zn
Vitamin K	Serum vitamin K Clotting time, prothrombin time, uncarboxylated Gla-proteins (PIVKA-II) (functional biomarkers)		
Vitamin B_12_	Serum or RBC vitamin B_12_ Serum holoTC2, plasma homocysteine, urine methylmalonate (functional biomarkers)		

Biomarkers of nutrient exposure are used to quantify the recent levels of consumed foods or nutrients in biological fluids. Such biomarkers can help stratifying individuals according to their consumption patterns such as whole grain^(99)^, fruit and vegetable, or meat and fish intake^(100)^. However, many of the biomarkers of exposure still need validation as a high variation between individuals is often observed^(100)^.

#### Show cases for classical nutrients in Switzerland: folic acid, vitamin D and iron

Although the Swiss population is generally considered ‘well nourished’ and mineral and vitamin deficiencies are not considered a major public health problem, no representative data exist on the prevalence and temporal development of nutritional deficiencies. Most of the data relies on small studies conducted in subgroups of the population.

Subgroups of the Swiss population may be at risk of folic acid deficiency, as a recent non representative survey identified 58 % of the study population with low plasma folate (14 nmol/l)^(10)^. In addition, severe vitamin D deficiency defined as 25-dihydroxycholecalciferol concentrations below 25 nmol/l was found in 34,2 % of 1’382 pregnant women attending prenatal care between 2012 and 2015, while low status 25-dihydroxycholecalciferol concentrations < 50 nmol/l was identified in 73 % of the sampled women^(9)^. While prevalence of anaemia is low, a screening study including 672 young women of reproductive age recruited from high schools, the University of Zürich and ETH Zürich resulted in an estimated prevalence of iron deficiency of 22·7 % (serum ferritin <15 µg/l)^(11)^. These studies are limited in scope, are not representative, and could be affected by sampling bias. Additionally, some of these analyses were conducted with convenience samples that were originally collected with different aims, not with the primary aim to assess nutritional status and its determinants, which can affect the outcome. Furthermore, the lack of a longitudinal component hampers in-depth analyses of predictors and associated factors. This is crucial for the identification of etiological patterns of nutrient deficiencies to design cost-effective and efficacious interventions.

#### Nutritional phenotyping

In order to expand abilities to capture nutrient-nutrient interdependencies and potentially to discover new biomarkers of nutrient status, the approach of nutritional phenotyping was introduced^(101)^. Nutritional phenotyping relates to the analytical possibilities to quantify a broad profile of nutrients and their related metabolites in biological fluids. This can be achieved thanks to the parallel use of complementary analytics including high pressure liquid-mass spectrometry, gas chromatography, inductively coupled mass spectrometry, and clinical chemistry. Within nutritional phenotyping, inductively coupled mass spectrometry is used to provide a quantitative profiling of elements enabling the so-called domain of ionomics. By analogy with metabolomics, ionomics aims at measuring the entire elemental composition of a living organism and its dynamics relative to genetic, physiological and metabolic variability^(102)^. Although less known and applied than metabolomics, ionomics has proven efficient in the study of element metabolism in isolated cells and in biological fluids^(103,104)^. Combined with recorded dietary information, nutritional phenotyping has the potential to study molecular interactions between the different nutrient families (amino acids, fatty acids, vitamins and minerals) while delivering information on classical biomarkers. This approach opens possibilities to identify nutrient patterns associated with various genetic, environmental, or phenotypic determinants that may help to identify novel nutrient status biomarkers.

A diverse range of pre-existing technologies, such as photography, microfluidics, wireless sensors and artificial intelligence may be combined and applied to nutrition research. These applications include the use of mobile phones to record and subsequently analyse dietary intake^(105)^, glucose sensors^(106)^ or microfluidic-based skin sensors measuring nutrients^(107)^.

## Precision nutrition

### Defining precision/personalised nutrition

The concept of personalised nutrition was put forward two decades ago in relation with the nutrigenomics approach promising to deliver personalised, health-directed, dietary guidelines, based on knowledge of the interactions between genes and diets^(108)^. This concept has evolved to integrate a more systematic approach of the interaction of diets with the human organism. It investigates gene–diet interaction, but also integrates different intrinsic datasets (epigenomics, transcriptomics, proteomics, metabolomics) at different structural levels of the human organisms (cells, organs, gut microbiota) under dynamic conditions, and considers environmental, extrinsic, factors such as physical activity^(109)^. The German Nutrition Society also proposes a model for personalised nutrition that goes beyond genetics to integrate phenotypic traits and the consumer^(110–113)^. Of note, a more detailed evaluation of the chemical composition of foods, for example, foodomics, is increasingly being recognised as an important component of nutrition research^(114)^. During the last years, nutrition researchers have integrated new technological tools such as wearable technologies^(115)^, Apps^(116)^ and photographic evaluation of food labels and dietary intake^(117,118)^. Cutting edge bioinformatics and biostatistical approaches consequently became essential tools for an efficient extraction of the information derived from modern nutritional studies^(119)^. Increasing the technology around nutrition research has led to the more recent concept of precision nutrition, although definitions for differentiating these two terms (i.e. personalised nutrition and precision nutrition) are still unclear^(120)^ and often used interchangeably^(121)^. The abbreviation PN (referring indistinctly to both personalised and precision nutrition) will consequently be used below to encompass this point. All in all, PN aims at using state-of-the-art, validated, analytical approaches to investigate the impact of nutrition, that is, nutrients, foods, and diets, on specific subgroups, even individual consumers. Given the level of detail accessible in modern nutritional studies, paradigms relying on discrete, homogeneous groups, may be less central and new data analysis tools such as principal component analysis (PCA) may be more promising, as the one-size-fits-all concept is this no longer valid. Some researchers push the boundaries of nutritional studies to the extreme case of single individuals in the case of n-of-1 studies^(122)^.

### Initiatives on precision nutrition

Several countries have recognised the strategic and public relevance of moving nutrition research to a PN. Among these, in the USA, the NIH has recently started, within its 2020–2030 Strategic Plan for NIH Nutrition research, a program awarding $170 million over 5 years for PN^(123,124)^. The awarded program will investigate 10’000 participants who are part of the NIH’s USA cohort with 1’000’000 participants. The NIH consortium includes several centres integrating clinical evaluations, dietary assessments, metabolomics analyses and clinical assay, gut microbiome analyses, as well as data modelling and bioinformatics. These tools will be used by the NIH to integrate lifestyle, biological, environmental, and social factors to develop eating recommendations for individual that improve overall health^(125)^.

At the European level, the Food4Me project was a precursor in 2012–2014 in developing and evaluating the personalised approach in nutrition research^(126)^, in particular by comparing three levels of personalisation based on diet, phenotype and genotype. This study concluded that PN-based advice achieves greater impact on dietary management by the participants^(127)^. The European Innovation Council has launched a call for a Pathfinder Challenge on precision nutrition. One of the objectives is to investigate causal relationships among diet, microbiome and glycans, with potential impact on personalizing human diet^(128)^.

### Analytical targets of personalised nutrition

Omics sciences at each level of the molecular flow of information in human cell are now integral parts of nutrition research, including nutritional cohorts. The analytical targets of PN not only investigate these molecular levels individually but also integrate them, and even goes beyond them, as presented in the following sections.

#### Genomes

A wide range of gene–diet interactions has been reported in the literature that impact on an equally broad range of phenotypic traits associated with metabolism^(129)^ and metabolic diseases^(130)^. Besides several examples of monogenetic nutrigenetic tests targeting clinical endpoints such blood pressure^(131)^, liver fibrosis^(132)^, myocardial infarction^(133)^, or obesity^(134)^, the combination of a range of polymorphisms involved in a particular phenotypic trait was superior to monogenetic tests in the context of obesity management^(135,136)^. Also, a genetic risk score based on SNPs associated with blood pressure may identify persons responsive to salt reduction^(137)^.

Genetic testing in nutritional counselling has benefits and limitations, highlighting the need for reproducing the reported study and, more importantly, identifying clinically useful gene–diet interactions^(138)^. Therefore, reaching clinical usefulness in PN certainly requests that PN goes beyond genetic tests and polygenetic scores, and integrates post-genetic molecular factors in its evaluation.

#### Epigenomes

The environment, including the diet^(139,140)^, is a key source of epigenetic modifications on DNA and histones. Nutriepigenetics is emerging as a key tool in PN. Dietary nutrients directly modulate DNA and even histones through the one-carbon pathway delivering methyl groups for epigenetic modifications. Work by Pembrey and colleagues suggested that access to calories of grandparents during puberty influence mortality of the grand-children though epigenetic modifications maintained across generations^(141)^. Although highly controversial due to the difficulty in going beyond mere statistical associations, this work found echoes in other studies investigating the association between access to food in 1945 during the Dutch Hunger winter of 1944–1945, epigenetic modifications and a range of cardiometabolic endpoints in the subsequent generations, including birth weight^(142)^. As such, nutrition is a key source of interindividual variability in human biology and epigenetic marks may contribute to the existence of metabotypes^(143)^. In particular, interindividual variation in DNA methylation is associated with obesity^(144)^ and epigenetic modifications of genes involved in gene–diet interactions were shown, in addition to genetics, to modulate the efficiency of weight loss programs^(145)^.

#### Metabotypes

The development of metabolomics in analytical chemistry has quickly found its application in nutrition research, evidently due to the primarily metabolic nature of human nutrition^(46)^. Metabolomics has become extremely popular in nutrition research thanks to its ability to study the quantitative expression of the real endpoints of the physiological regulatory processes, that is, the metabolites, in relation with health and disease outcomes, including nutrition-related metabolic risk factors for primary prevention^(101)^. Metabolomics is nowadays also a well-established approach to identify metabolic signatures associated with specific dietary intake from food groups to very specific foods such as dark chocolate^(146,147)^ or citrus fruits, proline betaine being one of the best characterised biomarkers of dietary intake^(148)^.

Recent research indicates that metabotypes associated with unfavourable metabolic status and incident disease occurrence are also characterised by diets low in vegetables, dairy products, and fibres, and highest intakes of total red and processed meat^(149)^. These findings open the door to the stratification of dietary guidance for consumers, based on their metabotypes^(150)^.

In that regard, defined as an extension of the pharmacokinetic concept in nutrition, nutrikinetics offers unique perspectives to study interindividual metabolic differences related to different food matrices^(151,152)^. It can capture interindividual differences in the response to nutrition and particularly to dietary phytochemicals that are metabolised by the gut microbiome, for example, microbiome co-metabolites hippuric acid, 4-hydroxyhippuric acid and 1,3-dihydroxyphenyl-2-O-sulfate as indicators of polyphenol-rich black tea consumption^(151)^. Because it enables to differentiate individuals based on their actual capability to process nutrients, also via the gut microbiome, nutrikinetics is foreseen as a powerful approach not only to infer host–microbiome nutrient interactions but also to objectively categorise individuals into fast/slow metabolisers for subsequent nutritional intervention trials.

#### Microbiome

Following the reduction in the cost of DNA sequencing and progresses in bioinformatics, the last decade has seen an explosion of research activities on the interactions between the gut microbiota and the human organism and, a few years later, on the impact of these interactions on health^(153)^. A characteristic of the gut microbiota is its large interindividual variability^(154)^ and the concept of enterotypes has emerged early in this field^(155)^. Diversity in the gut microbiota is intimately associated with variability in dietary intake. This implies that dietary regimens aimed at improving health, for example, the immune status^(156)^, should be tailored based on the gut microbiome^(157)^.

In essence, the direct interplay between diet and the microbiota, with intestinal microorganisms directly utilizing ingested nutrients, underscores the microbiota’s pivotal role in human nutrition. Therefore, alongside genetics and epigenetics, understanding the composition and functions of the gut microbiota is crucial to understand how diet impacts human health and disease.

#### Reference methods

Nutrition is characterised by subtle effects, which when added over long term, exert a high impact on health outcomes. Cohort studies offer the opportunity to deploy reference methods longitudinally to measure these subtle effects directly and with high precision and link them to the nutritional and environmental exposome. This allows for the discovery and identification of novel, previously undescribed associations, as well as the improved understanding of the effect of dietary patterns and components on the underlying human physiology.

Such methods currently include the use of stable or long-lived isotopes, such as the long-term monitoring of bone calcium balance via the use of 41-Ca isotopes^(158,159)^, stable iron isotopes to label body iron over the long term and to measure iron absorption and losses directly in the study participants^(160)^, or the measurement of energy expenditure with double labelled water^(161)^. A further, non-isotopic example for a high precision, established reference method is the utilisation of the CO rebreathing technique to assess blood volume and Hb mass, which improves the precision of the haemoglobin measurement substantially^(162)^. At term, PN is expected to make used of newly validated nutritional biomarkers to fuel the panel of reference methods available to researchers.

#### Non-Nutritional factors

The exposome refers to the totality of exposures from internal and external sources during the lifetime, including exposures to pollutants and other chemical and biological agents in addition to dietary compounds. The exposome is also contributed by psychosocial factors such as the socio-economic status^(163)^. Understanding the impact of nutrition on the health of the Swiss Cohort and Biobank proposed in the white paper of Probst–Hensch and colleagues^(60)^ thus requires that interactions between nutritional and non-nutritional elements of the exposomes and the human organism be taken into consideration. For illustration, the ability and/or willingness of participants in cohort studies to fully and adequately answer questionnaires, including dietary^(164)^ or socio-demographic^(165)^ questionnaires is subject to a large inter-individual variability that is influenced by factors such as older age, lower educational level, poorer health status and unhealthy lifestyle habits^(166)^. These biases need to be considered, for example, by imputing missing data or decreasing their occurrence by improving the response rate based on neuro-psychological tools. In addition, physical activity can modulate the interaction between diet and human metabolism, for example, for the impact of genes on body weight^(167)^. Also, the impact of the food environment on the intake of consumers at their residence, school, or workplace as well as their perception of this environment needs consideration^(168,169)^.

#### Towards nutritional systems biology

Can PN deliver on its promises?^(170)^ Penetrant phenotypic traits such as phenylketonuria are clear demonstrators of the potential of PN to translate knowledge into public health policies^(171)^. However, most chronic diseases are complex and the research field of PN consequently moves towards a combination of factors. The use of polygenic scores for the management of obesity illustrates this research direction although the clinical utility of this score has not been demonstrated yet^(172)^. Even traits with apparently clear causes cannot be pinpointed to isolated molecular events. For illustration, lactose intolerance not only involves polymorphisms upstream of the gene coding for lactase but is also modulated by epigenetic events as well as by the gut microbiota^(173)^.

The road to translate nutritional data into information that is relevant to the consumer’s health may thus well take the direction of systems biology and artificial intelligence^(174)^. Indeed, biomedical research is currently embracing the concept of systems biology, which combines structural, dynamics, modelling, and omics analytical approaches to further foster the translation of research from the laboratory to the bed^(175)^. Nutrition research follows this path by integrating elements such as the concept of the virtual patient^(176)^, whole-body models integrating metabolism, physiology and the gut microbiota^(177)^, phenotypic flexibility allowing for real-time evaluation of metabolism in response to a dietary challenge^(109)^, imaging techniques, such as functional MRI of the brain^(178)^, as well as combinations of multiple omics dataset^(179,180)^. For illustration, a retrospective cohort study used digital twin technology to reverse type 2 diabetes though precision nutrition^(181)^. This technology platform uses artificial intelligence to build a dynamic digital twin model of the patient with a broad range of data including, among others, clinical chemistry, dietary intake, exercise, and sleep recommendations.

## The Swiss cohort as a tool towards precision nutrition

A key point to establish the Swiss cohort will be to characterise the relevant health outcomes in relevant population groups^(182)^. The health outcome should be measured using appropriate clinical endpoints, including clear adjudication processes and validated risk factors to allow for the establishment of a high level of evidence for the investigated risk-outcome relationships. International efforts providing state-of-the-art insight into the assessment of risks, in particular dietary risks, will serve as basis for establishing the analytical strategy of the cohort^(3,8,183,184)^.

Determining the size of a cohort is a strategic issue that must consider, among others, scientific, economical, and logistic factors. For example, compared to other countries, Switzerland possesses a rather homogenous population when measured by socio-economic status; on the other hand, the geography (alpine region, plateau…) and culture (four national languages, high percentage of migrants, etc.) can be considered heterogenous. Estimating the size needed to have a representative cohort based on these factors is thus a complex task. Based on the experience gained internationally from existing large cohorts, Probst-Hensch and colleagues estimate in their White Paper that the Swiss Cohort & Biobank should enrol 100’000+ participants to account for the number and complexity of chronic diseases to be monitored and to allow for the identification of rare diseases^(60)^. Nutrition research should thus join force with medical research to add its arsenal of research tools to the analysis of the exposome of the Swiss Cohort & Biobank.

Translating nutritional research into information that will impact on the health of Swiss consumers thus requests that established research tools be combined with ground-breaking technologies. In addition to validated risk factors, new technologies and will also lead to the discovery of candidate biomarkers that will fuel the conduct of additional studies to validate them (see section on biomarkers below). The diet of the Swiss Cohort and Biobank will be linked to the phenotypic traits of the participants using state-of-the-art methodologies. The phenotypic traits include the medical history of the participants, their clinical chemistry, focusing on validated risk factors, as well as omics-analyses along the cellular flow of information (DNA, RNA, proteins, metabolites) in the biological samples collected in the cohort. In particular, blood cells will be used for genetic and epigenetic analyses; faecal water, serum/plasma, and urine for metabolomics, and the faeces for genomics analyses of the microbiome. The nutritional biomarkers identified in the cohort will provide information on (i) dietary intake, to complement classical dietary assessment, (ii) effect of dietary intake on the metabolism, to better evaluate the nutritional properties of the nutrients, foods or diets of interest, and finally (iii) susceptibility to dietary intake, to foster personalised nutrition^(185)^. The nutritional biomarkers will be validated according to the following criteria: plausibility, dose-response, time-response, robustness, reliability, stability, analytical performance, and inter-laboratory reproducibility^(186)^. Modern bioinformatics tools, including artificial intelligence, will be used to analyse associations in the triad diet–biomarkers–health.

Although longitudinal data in the Swiss cohort will provide some hints at mechanisms at play in the interaction of nutrients, foods and diets with the human organism and strong indication of causality can only be inferred from randomised controlled trial. Information gathered from the analysis of the triad diet-biomarker-health in the cohort will lead to the establishment of new nutritional hypotheses that will need to be tested in randomised controlled trials. These interventions could be conducted in subgroups of the Swiss cohort, using the so-called trials within cohorts design^(187)^ or in independent study groups. An analysis of the dietary behaviour of Swiss consumers has identified a dietary cluster that is specific to Switzerland (‘Swiss traditional’) and close to Western diet^(188)^. Developing a healthy and sustainable Swiss diet and demonstrating its benefits in an intervention study could serve as a proof a concept for the ability of Swiss nutritional research to translate knowledge into practice. To this end, understanding the Swiss consumer and developing the methodologies to motivate changes in dietary habits will be key.

The integration of high precision measurements in the cohort will allow for both the precise characterisation of selected aspects of nutritional status, the measurement its longitudinal development and the identification of relevant health associations to inform future interventions and to identify and discover novel risk factors and health associations. This will expand the knowledge base for nutritional sciences, promoting discoveries, but also, by employing reference methods, allowing for overcoming long-standing controversies in the nutrition field.

Although public health and PN appears at first to follow two opposite strategies with regards to the number of persons targeted by research, namely entire populations *v*. the individual, the public health nature of a Swiss cohort can indeed be fostered through PN by targeting large groups of consumers. PN advice can be targeted to consumer clusters with specific dietary patterns with a potential impact on health^(189)^ or to groups of citizens in living specific geographical areas or specific environments^(190)^. PN will thus contribute to an increased credibility of the nutritional sciences with the public and to an overall advancement of public health.

## References

[1] Semba RD (2012) The discovery of the vitamins. Int J Vitam Nutr Res 82, 310–315. 23798048 10.1024/0300-9831/a000124

[2] Mozaffarian D , Rosenberg I & Uauy R (2018) History of modern nutrition science-implications for current research, dietary guidelines, and food policy. BMJ 361, k2392. 10.1136/bmj.k2392 PMC5998735 29899124

[3] GBD 2017 Diet Collaborators (2019) Health effects of dietary risks in 195 countries, 1990–2017: a systematic analysis for the global burden of disease study 2017. Lancet 393, 1958–1972. 30954305 10.1016/S0140-6736(19)30041-8 PMC6899507

[4] Chong B , Jayabaskaran J , Kong G , et al. (2023) Trends and predictions of malnutrition and obesity in 204 countries and territories: an analysis of the global burden of disease study 2019. EClinicalMedicine 57, 101850. 36864983 10.1016/j.eclinm.2023.101850 PMC9971264

[5] WHO (2017) Ambition and Action in Nutrition 2016–2025. Geneva: WHO.

[6] Crippa M , Solazzo E , Guizzardi D , et al. (2021) Food systems are responsible for a third of global anthropogenic GHG emissions. Nat Food 2, 198–209. 37117443 10.1038/s43016-021-00225-9

[7] Intergovernmental Panel on Climate Change (2019) Special Report. Climate Change and Land. Geneva: Intergovernmental Panel on Climate Change.

[8] Willett W , Rockström J , Loken B , et al. (2019) Food in the anthropocene: the EAT-lancet commission on healthy diets from sustainable food systems. Lancet 393, 447–492. 30660336 10.1016/S0140-6736(18)31788-4

[9] Christoph P , Challande P , Raio L , et al. (2020) High prevalence of severe vitamin D deficiency during the first trimester in pregnant women in Switzerland and its potential contributions to adverse outcomes in the pregnancy. Swiss Med Wkly 150, w20238. 10.4414/smw.2020.20238 32502277

[10] Schüpbach R , Wegmüller R , Berguerand C , et al. (2017) Micronutrient status and intake in omnivores, vegetarians and vegans in Switzerland. Eur J Nutr 56, 283–293. 26502280 10.1007/s00394-015-1079-7

[11] Andersson M , Egli MI & Zimmermann MB (2010) Eisenmangel. Schweizer Z für Ernährungsmedizin 1, 6.

[12] Fischer L , Andersson M , Braegger C , et al. (2024) Iodine intake in the Swiss population 100 years after the introduction of iodised salt: a cross-sectional national study in children and pregnant women. Eur J Nutr 63, 573–587. 38141138 10.1007/s00394-023-03287-6 PMC10899291

[13] Agroscope (2022) 1. Swiss Nutrition Research Symposium - Sustainable Diet and Metabolic Health. Bern: Agroscope.

[14] Wietlisbach V , Paccaud F , Rickenbach M , et al. (1997) Trends in cardiovascular risk factors (1984–1993) in a Swiss region: results of three population surveys. Prev Med 26, 523–533. 9245675 10.1006/pmed.1997.0167

[15] Bopp M , Braun J , Faeh D , et al. (2010) Establishing a follow-up of the Swiss MONICA participants (1984–1993): record linkage with census and mortality data. BMC Public Health 10, 562. 20858236 10.1186/1471-2458-10-562 PMC2955001

[16] Gutzwiller F , Nater B & Martin J (1985) Community-based primary prevention of cardiovascular disease in Switzerland: methods and results of the national research program (NRP 1A). Prev Med 14, 482–491. 4070186 10.1016/0091-7435(85)90008-8

[17] Federal Statistical Office (2022) Schweizerische Gesundheitsbefragung 2022. Übersicht. Bern: Bundesamt für Gesundheit BAG.

[18] van der Horst K & Siegrist M (2011) Vitamin and mineral supplement users. Do they have healthy or unhealthy dietary behaviours? Appetite 57, 758–764. 21959200 10.1016/j.appet.2011.08.020

[19] Siegrist M & Hartmann C (2019) Impact of sustainability perception on consumption of organic meat and meat substitutes. Appetite 132, 196–202. 30322657 10.1016/j.appet.2018.09.016

[20] Morabia A , Bernstein M , Héritier S , et al. (1997) Community-based surveillance of cardiovascular risk factors in Geneva: methods, resulting distributions, and comparisons with other populations. Prev Med 26, 311–319. 9144755 10.1006/pmed.1997.0146

[21] Abreu D , Cardoso I , Gaspoz JM , et al. (2014) Trends in dietary intake in Switzerland, 1999–2009. Public Health Nutr 17, 479–485. 23425344 10.1017/S1368980013000207 PMC10282494

[22] Firmann M , Mayor V , Vidal PM , et al. (2008) The CoLaus study: a population-based study to investigate the epidemiology and genetic determinants of cardiovascular risk factors and metabolic syndrome. BMC Cardiovasc Disord 8, 6. 18366642 10.1186/1471-2261-8-6 PMC2311269

[23] Preisig M , Waeber G , Vollenweider P , et al. (2009) The PsyCoLaus study: methodology and characteristics of the sample of a population-based survey on psychiatric disorders and their association with genetic and cardiovascular risk factors. BMC Psychiatry 9, 9. 19292899 10.1186/1471-244X-9-9 PMC2667506

[24] CHUV (2023) CoLaus|PsyCoLaus. https://www.colaus-psycolaus.ch/ (accessed February 2024).

[25] Steinemann N , Grize L , Pons M , et al. (2018) Associations between dietary patterns and post-bronchodilation lung function in the SAPALDIA cohort. Respiration 95, 454–463. 29730665 10.1159/000488148

[26] Swiss Tropical amd Public Health Institute (1991) SAPALDIA Kohorte. Allschwil: Swiss Tropical amd Public Health Institute.

[27] Chatelan A , Beer-Borst S , Randriamiharisoa A , et al. (2017) Major differences in diet across three linguistic regions of Switzerland: results from the first national nutrition survey menuCH. Nutrients 9, 1163. 29068399 10.3390/nu9111163 PMC5707635

[28] Federal Food Safety and Veterinary Office (2023) Der Blick auf den Teller von Kindern und Jugendlichen in der Schweiz. https://www.blv.admin.ch/blv/de/home/lebensmittel-und-ernaehrung/forschung/gesundheitliche-risiken/ernaehrungsrisiken/menuch-kids.html (accessed February 2024)

[29] Gut A & Fröhli D (2022) Umfrage zu Nahrungsergänzungsmitteln. Bern: Bundesamt für Lebensmittelsicherheit und Veterinärwesen.

[30] Glatz N , Chappuis A , Conen D , et al. (2017) Associations of sodium, potassium and protein intake with blood pressure and hypertension in Switzerland. Swiss Med Wkly 147, w14411. 10.4414/smw.2017.14411 28322418

[31] Chappuis A , Bochud M , Glatz N , et al. (2011) Swiss Survey on Salt Intake: Main Results. Lausanne: Centre Hospitalier Universitaire Vaudois (CHUV).

[32] Federal Food Safety and Veterinary Office (2011) Assessing Salt Consumption in Switzerland. Bern: Federal Food Safety and Veterinary Office.

[33] Burri J , Haldimann M & Dudler V (2008) Selenium status of the Swiss population: assessment and change over a decade. J Trace Elem Med Biol 22, 112–119. 18565423 10.1016/j.jtemb.2007.11.002

[34] Federal Food Safety and Veterinary Office (2021) Biomonitoring – Selenium Status of the Population in Switzerland. Bern: Federal Food Safety and Veterinary Office.

[35] Federal Food Safety and Veterinary Office (2024) Monitoring the Iodine Supply in the Swiss Population. Bern: Federal Food Safety and Veterinary Office.

[36] Equey A , Berger MM , Gonseth-Nusslé S , et al. (2023) Association of plasma zinc levels with anti-SARS-CoV-2 IgG and IgA seropositivity in the general population: a case-control study. Clin Nutr 42, 972–986. 37130500 10.1016/j.clnu.2023.04.007 PMC10110932

[37] Hartmann C , Dohle S & Siegrist M (2013) Importance of cooking skills for balanced food choices. Appetite 65, 125–131. 23402717 10.1016/j.appet.2013.01.016

[38] Hagmann D , Siegrist M & Hartmann C (2019) Meat avoidance: motives, alternative proteins and diet quality in a sample of Swiss consumers. Public Health Nutr 22, 2448–2459. 31159899 10.1017/S1368980019001277 PMC10260614

[39] de Abreu D , Guessous I , Vaucher J , et al. (2013) Low compliance with dietary recommendations for food intake among adults. Clin Nutr 32, 783–788. 23260749 10.1016/j.clnu.2012.11.022

[40] Vlaanderen J , de Hoogh K , Hoek G , et al. (2021) Developing the building blocks to elucidate the impact of the urban exposome on cardiometabolic-pulmonary disease: the EU EXPANSE project. Environ Epidemiol 5, e162. 34414346 10.1097/EE9.0000000000000162 PMC8367039

[41] Pestoni G , Krieger JP , Sych JM , et al. (2019) Cultural differences in diet and determinants of diet quality in Switzerland: results from the national nutrition survey menuCH. Nutrients 11, 126. 30634520 10.3390/nu11010126 PMC6357532

[42] Office FS (2021) Schweizerische Gesundheitsbefragung. https://www.bfs.admin.ch/bfs/de/home/statistiken/gesundheit/erhebungen/sgb.html (accessed February 2024).

[43] Brombach C , Haefeli D , Bartsch S , et al. (2014) Ernährungsmuster im verlauf von drei generationen: gibt es inter- und intraindividuelle unterschiede? Internationaler Arbeitskreis für Kulturforschung Essens Mitteilungen 21, 11.

[44] Brombach C , Bartsch S & Gertrud Winkler G (2015) Ernährungsverhalten im verlauf von drei generationen. Schweizer Z für Ernährungsmedizin 5, 20–25.

[45] Subar AF , Kipnis V , Troiano RP , et al. (2003) Using intake biomarkers to evaluate the extent of dietary misreporting in a large sample of adults: the OPEN study. Am J Epidemiol 158, 1–13. 12835280 10.1093/aje/kwg092

[46] Ulaszewska MM , Weinert CH , Trimigno A , et al. (2019) Nutrimetabolomics: an integrative action for metabolomic analyses in human nutritional studies. Mol Nutr Food Res 63, e1800384. 30176196 10.1002/mnfr.201800384

[47] Popoola AA , Frediani JK , Hartman TJ , et al. (2023) Mitigating underreported error in food frequency questionnaire data using a supervised machine learning method and error adjustment algorithm. BMC Med Inform Decis Mak 23, 178. 37689645 10.1186/s12911-023-02262-9 PMC10492312

[48] Suter F , Pestoni G , Sych J , et al. (2023) Alcohol consumption: context and association with mortality in Switzerland. Eur J Nutr 62, 1331–1344. 36564527 10.1007/s00394-022-03073-w PMC10030531

[49] Suter F , Karavasiloglou N , Braun J , et al. (2023) Is following a cancer-protective lifestyle linked to reduced cancer mortality risk? Int J Public Health 68, 1605610. 36866000 10.3389/ijph.2023.1605610 PMC9970999

[50] Krieger JP , Pestoni G , Frehner A , et al. (2020) Combining recent nutritional data with prospective cohorts to quantify the impact of modern dietary patterns on disability-adjusted life years: a feasibility study. Nutrients 12, 833. 32245025 10.3390/nu12030833 PMC7146619

[51] Krieger JP , Cabaset S , Pestoni G , et al. (2018) Dietary patterns are associated with cardiovascular and cancer mortality among Swiss adults in a census-linked cohort. Nutrients 10, 313. 29518908 10.3390/nu10030313 PMC5872731

[52] Rohrmann S , Braun J , Bopp M , et al. (2013) Inverse association between circulating vitamin D and mortality--dependent on sex and cause of death? Nutr Metab Cardiovasc Dis 23, 960–966. 24095147 10.1016/j.numecd.2013.05.005

[53] Lohse T , Faeh D , Bopp M , et al. (2016) Adherence to the cancer prevention recommendations of the world cancer research fund/American institute for cancer research and mortality: a census-linked cohort. Am J Clin Nutr 104, 678–685. 27488239 10.3945/ajcn.116.135020

[54] Federal Food Safety and Veterinary Office (2023) Swiss Food Composition Database. Bern: Federal Food Safety and Veterinary Office.

[55] Brunner TA , van der Horst K & Siegrist M (2010) Convenience food products. Drivers for consumption. Appetite 55, 498–506. 20832437 10.1016/j.appet.2010.08.017

[56] Sob C , Siegrist M , Hagmann D , et al. (2021) A longitudinal study examining the influence of diet-related compensatory behavior on healthy weight management. Appetite 156, 104975. 32966848 10.1016/j.appet.2020.104975

[57] Long D , Mackenbach JP , Klokgieters S , et al. (2023) Widening educational inequalities in mortality in more recent birth cohorts: a study of 14 European countries. J Epidemiol Community Health 77, 400–408. 37094941 10.1136/jech-2023-220342 PMC10176379

[58] Vaccarella S , Georges D , Bray F , et al. (2023) Socioeconomic inequalities in cancer mortality between and within countries in Europe: a population-based study. Lancet Reg Health Eur 25, 100551. 36818237 10.1016/j.lanepe.2022.100551 PMC9929598

[59] Baggio S , Abarca M , Bodenmann P , et al. (2015) Early childhood caries in Switzerland: a marker of social inequalities. BMC Oral Health 15, 82. 26198542 10.1186/s12903-015-0066-y PMC4511018

[60] Probst-Hensch N , Bochud M , Chiolero A , et al. (2022) Swiss cohort & biobank - the white paper. Public Health Rev 43, 1605660. 36619237 10.3389/phrs.2022.1605660 PMC9817110

[61] Kirkpatrick SI , Reedy J , Butler EN , et al. (2014) Dietary assessment in food environment research: a systematic review. Am J Prev Med 46, 94–102. 24355678 10.1016/j.amepre.2013.08.015 PMC4558887

[62] Subar AF , Freedman LS , Tooze JA , et al. (2015) Addressing current criticism regarding the value of self-report dietary data. J Nutr 145, 2639–2645. 26468491 10.3945/jn.115.219634 PMC4656907

[63] Kirkpatrick SI , Vanderlee L , Raffoul A , et al. (2017) Self-report dietary assessment tools used in Canadian research: a scoping review. Adv Nutr 8, 276–289. 28298272 10.3945/an.116.014027 PMC5347105

[64] EFSA (2014) Guidance on the EU menu methodology. EFSA J 12, 3944.

[65] Harttig U , Haubrock J , Knüppel S , et al. (2011) The MSM program: web-based statistics package for estimating usual dietary intake using the multiple source method. Eur J Clin Nutr 65, S87–91. 21731011 10.1038/ejcn.2011.92

[66] Carter MC , Albar SA , Morris MA , et al. (2015) Development of a UK online 24-h dietary assessment tool: myfood24. Nutrients 7, 4016–4032. 26024292 10.3390/nu7064016 PMC4488770

[67] Subar AF , Kirkpatrick SI , Mittl B , et al. (2012) The automated self-administered 24-hour dietary recall (ASA24): a resource for researchers, clinicians, and educators from the national cancer institute. J Acad Nutr Diet 112, 1134–1137. 22704899 10.1016/j.jand.2012.04.016 PMC3721511

[68] Federal Food Safety and Veterinary Office (2022) La méthode ASA24 reconstituera-elle le puzzle de la consommation alimentaire ? https://www.blv.admin.ch/blv/fr/home/lebensmittel-und-ernaehrung/forschung/gesundheitliche-risiken/ernaehrungsrisiken/asa24.html (accessed February 2024).

[69] Zuppinger C , Taffé P , Burger G , et al. (2022) Performance of the digital dietary assessment tool MyFoodRepo. Nutrients 14, 635. 35276994 10.3390/nu14030635 PMC8838173

[70] Pannen ST , Gassmann R , Vorburger R , et al. (2023) Development of a multilingual web-based food frequency questionnaire for adults in Switzerland. Nutrients 15, 4359. 37892434 10.3390/nu15204359 PMC10610353

[71] González-Domínguez R , González-Domínguez Á , Sayago A , et al. (2020) Recommendations and best practices for standardizing the pre-analytical processing of blood and urine samples in metabolomics. Metabolites 10, 229. 32503183 10.3390/metabo10060229 PMC7344701

[72] Williamson S , Munro C , Pickler R , et al. (2012) Comparison of biomarkers in blood and saliva in healthy adults. Nurs Res Pract 2012, 246178. 22619709 10.1155/2012/246178 PMC3350846

[73] Bi H , Guo Z , Jia X , et al. (2020) The key points in the pre-analytical procedures of blood and urine samples in metabolomics studies. Metabolomics 16, 68. 32451742 10.1007/s11306-020-01666-2

[74] González-Gross M , Breidenassel C , Gómez-Martínez S , et al. (2008) Sampling and processing of fresh blood samples within a European multicenter nutritional study: evaluation of biomarker stability during transport and storage. Int J Obes (Lond) 32, S66–75. 19011656 10.1038/ijo.2008.185

[75] Locatelli M , Tartaglia A , D’Ambrosio F , et al. (2020) Biofluid sampler: A new gateway for mail-in-analysis of whole blood samples. J Chromatogr B Analyt Technol Biomed Life Sci 1143, 122055. 10.1016/j.jchromb.2020.122055 32172173

[76] Hoffman MSF , McKeage JW , Xu J , et al. (2023) Minimally invasive capillary blood sampling methods. Expert Rev Med Devices 20, 5–16. 36694960 10.1080/17434440.2023.2170783

[77] Zimmer JS , Christianson CD , Johnson CJ , et al. (2013) Recent advances in the bioanalytical applications of dried matrix spotting for the analysis of drugs and their metabolites. Bioanalysis 5, 2581–2588. 24138629 10.4155/bio.13.214

[78] Wang Y , Li R , Shu W , et al. (2024) Designed nanomaterials-assisted proteomics and metabolomics analysis for *i*n vitro diagnosis. Small Methods 8, e2301192. 37922520 10.1002/smtd.202301192

[79] De Biasi S , Gigan JP , Borella R , et al. (2024) Cell metabolism: functional and phenotypic single cell approaches. Methods Cell Biol 186, 151–187. 38705598 10.1016/bs.mcb.2024.02.024

[80] Garde AH , Hansen AM , Kristiansen J , et al. (2004) Comparison of uncertainties related to standardization of urine samples with volume and creatinine concentration. Ann Occup Hyg 48, 171–179. 14990438 10.1093/annhyg/meh019

[81] Witte EC , Lambers Heerspink HJ , de Zeeuw D , et al. (2009) First morning voids are more reliable than spot urine samples to assess microalbuminuria. J Am Soc Nephrol 20, 436–443. 19092125 10.1681/ASN.2008030292 PMC2637059

[82] Soldi LR , Maltos AL , da Cunha DF , et al. (2018) Correlation between first morning single void and 24-hour urines: the reliability to quantify Niacin status. Med Sci Monit Basic Res 24, 206–209. 30473581 10.12659/MSMBR.910087 PMC6282649

[83] Fernández-Peralbo MA & Luque de Castro MD (2012) Preparation of urine samples prior to targeted or untargeted metabolomics mass-spectrometry analysis. TrAC, Trends Anal Chem 41, 75–85.

[84] De Wardener HE (1985) Tests of glomerular functional integrity and proteinuria. In The Kidney. Edinburgh: Churchill Livingstone.

[85] Harris SA , Purdham JT , Corey PN , et al. (2000) An evaluation of 24-hour urinary creatinine excretion for use in identification of incomplete urine collections and adjustment of absorbed dose of pesticides. Aihaj 61, 649–657. 11071416 10.1080/15298660008984574

[86] Drummer OH (2008) Introduction and review of collection techniques and applications of drug testing of oral fluid. Ther Drug Monit 30, 203–206. 18367981 10.1097/FTD.0b013e3181679015

[87] Bellagambi FG , Lomonaco T , Salvo P , et al. (2020) Saliva sampling: methods and devices. An overview. TrAC, Trends Anal Chem 124, 115781.

[88] Battino M , Ferreiro MS , Gallardo I , et al. (2002) The antioxidant capacity of saliva. J Clin Periodontol 29, 189–194. 11940135 10.1034/j.1600-051x.2002.290301x.x

[89] Lusa Cadore E , Lhullier FL , Arias Brentano M , et al. (2009) Salivary hormonal responses to resistance exercise in trained and untrained middle-aged men. J Sports Med Phys Fitness 49, 301–307. 19861937

[90] Palanisamy V , Sharma S , Deshpande A , et al. (2010) Nanostructural and transcriptomic analyses of human saliva derived exosomes. PLoS One 5, e8577. 20052414 10.1371/journal.pone.0008577 PMC2797607

[91] Jacobs N , Nicolson NA , Derom C , et al. (2005) Electronic monitoring of salivary cortisol sampling compliance in daily life. Life Sci 76, 2431–2443. 15763075 10.1016/j.lfs.2004.10.045

[92] Votruba SB , Shaw PA , Oh EJ , et al. (2019) Associations of plasma, RBCs, and hair carbon and nitrogen isotope ratios with fish, meat, and sugar-sweetened beverage intake in a 12-wk inpatient feeding study. Am J Clin Nutr 110, 1306–1315. 31515553 10.1093/ajcn/nqz208 PMC6885477

[93] Wessells KR , Brown KH , Arnold CD , et al. (2021) Plasma and nail zinc concentrations, but not hair zinc, respond positively to two different forms of preventive zinc supplementation in young Laotian children: a randomized controlled trial. Biol Trace Elem Res 199, 442–452. 32356207 10.1007/s12011-020-02163-2 PMC7746564

[94] Ahn S , Hwang JE , Kim YJ , et al. (2024) Examination of the utility of skin carotenoid status in estimating dietary intakes of carotenoids and fruits and vegetables: a randomized, parallel-group, controlled feeding trial. Nutr 119, 112304. 10.1016/j.nut.2023.112304 38154397

[95] den Besten G , van Eunen K , Groen AK , et al. (2013) The role of short-chain fatty acids in the interplay between diet, gut microbiota, and host energy metabolism. J Lipid Res 54, 2325–2340. 23821742 10.1194/jlr.R036012 PMC3735932

[96] Deda O , Gika HG , Wilson ID , et al. (2015) An overview of fecal sample preparation for global metabolic profiling. J Pharm Biomed Anal 113, 137–150. 25812436 10.1016/j.jpba.2015.02.006

[97] Picó C , Serra F , Rodríguez AM , et al. (2019) Biomarkers of nutrition and health: new tools for new approaches. Nutrients 11, 1092. 31100942 10.3390/nu11051092 PMC6567133

[98] Berger MM , Shenkin A , Schweinlin A , et al. (2022) ESPEN micronutrient guideline. Clin Nutr 41, 1357–1424. 35365361 10.1016/j.clnu.2022.02.015

[99] Ross AB , Bourgeois A , Macharia HN , et al. (2012) Plasma alkylresorcinols as a biomarker of whole-grain food consumption in a large population: results from the WHOLEheart intervention study. Am J Clin Nutr 95, 204–211. 22170369 10.3945/ajcn.110.008508 PMC3592483

[100] Dragsted LO (2010) Biomarkers of meat intake and the application of nutrigenomics. Meat Sci 84, 301–307. 20374789 10.1016/j.meatsci.2009.08.028

[101] Rezzi S , Collino S , Goulet L , et al. (2013) Metabonomic approaches to nutrient metabolism and future molecular nutrition. TrAC, Trends Anal Chem 52, 112–119.

[102] Salt DE , Baxter I & Lahner B (2008) Ionomics and the study of the plant ionome. Annu Rev Plant Biol 59, 709–733. 18251712 10.1146/annurev.arplant.59.032607.092942

[103] Konz T , Monnard C , Restrepo MR , et al. (2020) Multielemental analysis of low-volume samples reveals cancer-specific profile in serum and sorted immune cells. Anal Chem 92, 8750–8758. 32460479 10.1021/acs.analchem.9b05643

[104] Konz T , Santoro A , Goulet L , et al. (2018) Sex-specific associations of blood-based nutrient profiling with body composition in the elderly. Front Physiol 9, 1935. 30733685 10.3389/fphys.2018.01935 PMC6353856

[105] Mohanty SP , Singhal G , Scuccimarra EA , et al. (2022) The food recognition benchmark: using deep learning to recognize food in images. Front Nutr 9, 875143. 35600815 10.3389/fnut.2022.875143 PMC9121091

[106] Boscari F , Vettoretti M , Amato AML , et al. (2021) Comparing the accuracy of transcutaneous sensor and 90-day implantable glucose sensor. Nutr Metab Cardiovasc Dis 31, 650–657. 33594987 10.1016/j.numecd.2020.09.006

[107] Kim J , Wu Y , Luan H , et al. (2022) A skin-interfaced, miniaturized microfluidic analysis and delivery system for colorimetric measurements of nutrients in sweat and supply of vitamins through the skin. Adv Sci (Weinh) 9, e2103331. 34747140 10.1002/advs.202103331 PMC8805554

[108] Kaput J , Ordovas JM , Ferguson L , et al. (2005) The case for strategic international alliances to harness nutritional genomics for public and personal health. Br J Nutr 94, 623–632. 16277761 10.1079/bjn20051585

[109] van Ommen B , van den Broek T , de Hoogh I , et al. (2017) Systems biology of personalized nutrition. Nutr Rev 75, 579–599. 28969366 10.1093/nutrit/nux029 PMC5914356

[110] German Nutrition Society (2022) Personalisierte Ernährung Neu Gedacht. Bonn: Deutsche Gesellschaft für Ernährung e. V.

[111] Holzapfel C , Waldenberger M , Lorkowski S , et al. (2022) Genetics and epigenetics in personalized nutrition: evidence, expectations, and experiences. Mol Nutr Food Res 66, e2200077. 35770348 10.1002/mnfr.202200077

[112] Simon MC , Sina C , Ferrario PG , et al. (2023) Gut microbiome analysis for personalized nutrition: the state of science. Mol Nutr Food Res 67, e2200476. 36424179 10.1002/mnfr.202200476

[113] Renner B , Buyken AE , Gedrich K , et al. (2023) Perspective: a conceptual framework for adaptive personalized nutrition advice systems (APNASs). Adv Nutr 14, 983–994. 37419418 10.1016/j.advnut.2023.06.009 PMC10509404

[114] Bordoni A & Capozzi F (2014) Foodomics for healthy nutrition. Curr Opin Clin Nutr Metab Care 17, 418–424. 25010544 10.1097/MCO.0000000000000089

[115] Shi Z , Li X , Shuai Y , et al. (2022) The development of wearable technologies and their potential for measuring nutrient intake: towards precision nutrition. Nutr Bull 47, 388–406. 36134894 10.1111/nbu.12581

[116] DiFilippo KN , Huang WH , Andrade JE , et al. (2015) The use of mobile apps to improve nutrition outcomes: a systematic literature review. J Telemed Telecare 21, 243–253. 25680388 10.1177/1357633X15572203

[117] Lazzari G , Jaquet Y , Kebaili DJ , et al. (2018) FoodRepo: an open food repository of barcoded food products. Front Nutr 5, 57. 30023359 10.3389/fnut.2018.00057 PMC6040205

[118] Salathé M , Bengtsson L , Bodnar TJ , et al. (2012) Digital epidemiology. PLoS Comput Biol 8, e1002616. 22844241 10.1371/journal.pcbi.1002616 PMC3406005

[119] Dang S & Vialaneix N (2018) Cutting edge bioinformatics and biostatistics approaches are bringing precision medicine and nutrition to a new era. Lifestyle Genom 11, 73–76. 30472706 10.1159/000494131

[120] Ferguson LR , De Caterina R , Görman U , et al. (2016) Guide and position of the international society of nutrigenetics/nutrigenomics on personalised nutrition: part 1 - fields of precision nutrition. J Nutrigenet Nutrigenomics 9, 12–27. 27169401 10.1159/000445350

[121] Blaak EE , Roche HM & Afman LA (2021) Editorial: personalized nutrition. Front Nutr 8, 669307. 33842527 10.3389/fnut.2021.669307 PMC8027071

[122] Kaput J (2021) Developing the pathway to personalized health: the potential of N-of-1 studies for personalizing nutrition. J Nutr 151, 2863–2864. 34293136 10.1093/jn/nxab243

[123] Kaiser J (2021) NIH’s ‘precision nutrition’ bet aims for individualized diets. Sci 371, 552. 10.1126/science.371.6529.552 33542117

[124] Lee BY , Ordovás JM , Parks EJ , et al. (2022) Research gaps and opportunities in precision nutrition: an NIH workshop report. Am J Clin Nutr 116, 1877–1900. 36055772 10.1093/ajcn/nqac237 PMC9761773

[125] NIH (2022) NIH awards $170 million for precision nutrition study. https://www.nih.gov/news-events/news-releases/nih-awards-170-million-precision-nutrition-study (accessed February 2024).

[126] Stewart-Knox B , Rankin A , Kuznesof S , et al. (2015) Promoting healthy dietary behaviour through personalised nutrition: technology push or technology pull? Proc Nutr Soc 74, 171–176. 25342299 10.1017/S0029665114001529

[127] Livingstone KM , Celis-Morales C , Navas-Carretero S , et al. (2021) Personalised nutrition advice reduces intake of discretionary foods and beverages: findings from the Food4Me randomised controlled trial. Int J Behav Nutr Phys Act 18, 70. 34092234 10.1186/s12966-021-01136-5 PMC8183081

[128] European Commission (2023) EIC Pathfinder Challenge: Precision Nutrition. –https://ec.europa.eu/info/funding-tenders/opportunities/portal/screen/opportunities/topic-details/horizon-eic-2023-pathfinderchallenges-01–03 (accessed February 2024).

[129] Floris M , Cano A , Porru L , et al. (2020) Direct-to-consumer nutrigenetics testing: an overview. Nutrients 12, 566. 32098227 10.3390/nu12020566 PMC7071525

[130] Barrea L , Annunziata G , Bordoni L , et al. (2020) Nutrigenetics-personalized nutrition in obesity and cardiovascular diseases. Int J Obes Suppl 10, 1–13. 32714508 10.1038/s41367-020-0014-4 PMC7371677

[131] Poch E , González D , Giner V , et al. (2001) Molecular basis of salt sensitivity in human hypertension. Evaluation of renin-angiotensin-aldosterone system gene polymorphisms. Hypertens 38, 1204–1209. 10.1161/hy1101.099479 11711524

[132] Vilar-Gomez E , Pirola CJ , Sookoian S , et al. (2021) Impact of the association between PNPLA3 genetic variation and dietary intake on the risk of significant fibrosis in patients with NAFLD. Am J Gastroenterol 116, 994–1006. 33306506 10.14309/ajg.0000000000001072 PMC8087619

[133] Cornelis MC , El-Sohemy A , Kabagambe EK , et al. (2006) Coffee, CYP1A2 genotype, and risk of myocardial infarction. Jama 295, 1135–1141. 16522833 10.1001/jama.295.10.1135

[134] Corella D , Peloso G , Arnett DK , et al. (2009) APOA2, dietary fat, and body mass index: replication of a gene–diet interaction in 3 independent populations. Arch Intern Med 169, 1897–1906. 19901143 10.1001/archinternmed.2009.343 PMC2874956

[135] Kalantari N , Doaei S , Keshavarz-Mohammadi N , et al. (2016) Review of studies on the fat mass and obesity-associated (FTO) gene interactions with environmental factors affecting on obesity and its impact on lifestyle interventions. ARYA Atheroscler 12, 281–290. 28607568 PMC5455327

[136] Cha S , Kang JH , Lee JH , et al. (2018) Impact of genetic variants on the individual potential for body fat loss. Nutrients 10, 266. 29495392 10.3390/nu10030266 PMC5872684

[137] Nierenberg JL , Li C , He J , et al. (2017) Blood pressure genetic risk score predicts blood pressure responses to dietary sodium and potassium: the GenSalt study (genetic epidemiology network of salt sensitivity). Hypertens 70, 1106–1112. 10.1161/HYPERTENSIONAHA.117.10108 PMC6443381 28993450

[138] van der Horst K , von Meyenn F , Rezzi S , et al. (2022) Sind nutrigenetiche tests bereit für den alltag? Schweizer Z für Ernährungsmedizin 1, 4.

[139] Delage B & Dashwood RH (2008) Dietary manipulation of histone structure and function. Annu Rev Nutr 28, 347–366. 18598138 10.1146/annurev.nutr.28.061807.155354 PMC2737739

[140] Alegría-Torres JA , Baccarelli A & Bollati V (2011) Epigenetics and lifestyle. Epigenomics 3, 267–277. 22122337 10.2217/epi.11.22 PMC3752894

[141] Pembrey ME , Bygren LO , Kaati G , et al. (2006) Sex-specific, male-line transgenerational responses in humans. Eur J Hum Genet 14, 159–166. 16391557 10.1038/sj.ejhg.5201538

[142] Lumey LH & Stein AD (1997) Offspring birth weights after maternal intrauterine undernutrition: a comparison within sibships. Am J Epidemiol 146, 810–819. 9384201 10.1093/oxfordjournals.aje.a009198

[143] Hellbach F , Baumeister SE , Wilson R , et al. (2022) Association between usual dietary intake of food groups and DNA methylation and effect modification by metabotype in the KORA FF4 cohort. Life (Basel) 12, 1064. 35888152 10.3390/life12071064 PMC9318948

[144] Kühnen P , Handke D , Waterland RA , et al. (2016) Interindividual variation in DNA methylation at a putative POMC metastable epiallele is associated with obesity. Cell Metab 24, 502–509. 27568547 10.1016/j.cmet.2016.08.001

[145] Sun D , Heianza Y , Li X , et al. (2018) Genetic, epigenetic and transcriptional variations at NFATC2IP locus with weight loss in response to diet interventions: the POUNDS lost trial. Diabetes Obes Metab 20, 2298–2303. 29693310 10.1111/dom.13333 PMC6105429

[146] Guasch-Ferré M , Bhupathiraju SN & Hu FB (2018) Use of metabolomics in improving assessment of dietary intake. Clin Chem 64, 82–98. 29038146 10.1373/clinchem.2017.272344 PMC5975233

[147] Rezzi S , Ramadan Z , Fay LB , et al. (2007) Nutritional metabonomics: applications and perspectives. J Proteome Res 6, 513–525. 17269708 10.1021/pr060522z

[148] Heinzmann SS , Brown IJ , Chan Q , et al. (2010) Metabolic profiling strategy for discovery of nutritional biomarkers: proline betaine as a marker of citrus consumption. Am J Clin Nutr 92, 436–443. 20573794 10.3945/ajcn.2010.29672 PMC2904656

[149] Riedl A , Hillesheim E , Wawro N , et al. (2020) Evaluation of the metabotype concept identified in an Irish population in the German KORA cohort study. Mol Nutr Food Res 64, e1900918. 32048458 10.1002/mnfr.201900918

[150] Hillesheim E , Ryan MF , Gibney E , et al. (2020) Optimisation of a metabotype approach to deliver targeted dietary advice. Nutr Metab (Lond) 17, 82. 33005208 10.1186/s12986-020-00499-z PMC7523294

[151] van Velzen EJ , Westerhuis JA , van Duynhoven JP , et al. (2009) Phenotyping tea consumers by nutrikinetic analysis of polyphenolic end-metabolites. J Proteome Res 8, 3317–3330. 19374449 10.1021/pr801071p

[152] van Duynhoven JPM , van Velzen EJJ , Westerhuis JA , et al. (2012) Nutrikinetics: concept, technologies, applications, perspectives. Trends Food Sci Technol 26, 4–13.

[153] Flint HJ , Scott KP , Louis P , et al. (2012) The role of the gut microbiota in nutrition and health. Nat Rev Gastroenterol Hepatol 9, 577–589. 22945443 10.1038/nrgastro.2012.156

[154] Leeming ER , Johnson AJ , Spector TD , et al. (2019) Effect of diet on the gut microbiota: rethinking intervention duration. Nutrients 11, 2862. 31766592 10.3390/nu11122862 PMC6950569

[155] Arumugam M , Raes J , Pelletier E , et al. (2011) Enterotypes of the human gut microbiome. Nat 473, 174–180. 10.1038/nature09944 PMC3728647 21508958

[156] Wastyk HC , Fragiadakis GK , Perelman D , et al. (2021) Gut-microbiota-targeted diets modulate human immune status. Cell 184, 4137–4153.e4114. 34256014 10.1016/j.cell.2021.06.019 PMC9020749

[157] Johnson AJ , Vangay P , Al-Ghalith GA , et al. (2019) Daily sampling reveals personalized diet-microbiome associations in humans. Cell Host Microbe 25, 789–802.e785. 31194939 10.1016/j.chom.2019.05.005

[158] Hodges JK , Maiz M , Lachcik PJ , et al. (2023) Moderate consumption of freeze-dried blueberry powder increased net bone calcium retention compared with no treatment in healthy postmenopausal women: a randomized crossover trial. Am J Clin Nutr 118, 382–390. 37269909 10.1016/j.ajcnut.2023.05.033 PMC10447493

[159] Denk E , Hillegonds D , Hurrell RF , et al. (2007) Evaluation of 41calcium as a new approach to assess changes in bone metabolism: effect of a bisphosphonate intervention in postmenopausal women with low bone mass. J Bone Miner Res 22, 1518–1525. 17576167 10.1359/jbmr.070617

[160] Speich C , Mitchikpè CES , Cercamondi CI , et al. (2021) Direct assessment of body iron balance in women with and without iron supplementation using a long-term isotope dilution method in Benin and Switzerland. Am J Clin Nutr 113, 1657–1669. 33693464 10.1093/ajcn/nqaa433

[161] Speakman JR , de Jong JMA , Sinha S , et al. (2023) Total daily energy expenditure has declined over the past three decades due to declining basal expenditure, not reduced activity expenditure. Nat Metab 5, 579–588. 37100994 10.1038/s42255-023-00782-2 PMC10445668

[162] Schmidt W & Prommer N (2005) The optimised CO-rebreathing method: a new tool to determine total haemoglobin mass routinely. Eur J Appl Physiol 95, 486–495. 16222540 10.1007/s00421-005-0050-3

[163] Miller GW & Jones DP (2014) The nature of nurture: refining the definition of the exposome. Toxicol Sci 137, 1–2. 24213143 10.1093/toxsci/kft251 PMC3871934

[164] Ahn Y , Paik HY & Ahn YO (2006) Item non-responses in mailed food frequency questionnaires in a Korean male cancer cohort study. Asia Pac J Clin Nutr 15, 170–177. 16672200

[165] Andreeva VA , Galan P , Julia C , et al. (2014) Assessment of response consistency and respective participant profiles in the internet-based NutriNet-Santé cohort. Am J Epidemiol 179, 910–916. 24521560 10.1093/aje/kwt431 PMC3969533

[166] Tsiampalis T & Panagiotakos DB (2020) Missing-data analysis: socio- demographic, clinical and lifestyle determinants of low response rate on self- reported psychological and nutrition related multi- item instruments in the context of the ATTICA epidemiological study. BMC Med Res Methodol 20, 148. 32513107 10.1186/s12874-020-01038-3 PMC7281925

[167] Muhammad HFL , Sulistyoningrum DC , Huriyati E , et al. (2021) The interaction between energy intake, physical activity and UCP2 -866G/A gene variation on weight gain and changes in adiposity: an Indonesian nutrigenetic cohort (INDOGENIC). Br J Nutr 125, 611–617. 32746947 10.1017/S0007114520003104

[168] Myers CA (2023) Impact of the neighborhood food environment on dietary intake and obesity: a review of the recent literature. Curr Diab Rep 23, 371–386. 38008848 10.1007/s11892-023-01529-9

[169] Pineda E , Bascunan J & Sassi F (2021) Improving the school food environment for the prevention of childhood obesity: what works and what doesn’t. Obes Rev 22, e13176. 33462933 10.1111/obr.13176

[170] Ferrario PG , Watzl B , Møller G , et al. (2021) What is the promise of personalised nutrition? J Nutr Sci 10, e23. 33996036 10.1017/jns.2021.13 PMC8080179

[171] Lichter-Konecki U & Vockley J (2019) Phenylketonuria: current treatments and future developments. Drugs 79, 495–500. 30864096 10.1007/s40265-019-01079-z

[172] Höchsmann C , Yang S , Ordovás JM , et al. (2023) The personalized nutrition study (POINTS): evaluation of a genetically informed weight loss approach, a randomized clinical trial. Nat Commun 14, 6321. 37813841 10.1038/s41467-023-41969-1 PMC10562431

[173] Porzi M , Burton-Pimentel KJ , Walther B , et al. (2021) Development of personalized nutrition: applications in lactose intolerance diagnosis and management. Nutrients 13, 1503. 33946892 10.3390/nu13051503 PMC8145768

[174] Ferrario PG & Gedrich K (2024) Machine learning and personalized nutrition: a promising liaison? Eur J Clin Nutr 78, 74–76. 37833568 10.1038/s41430-023-01350-3 PMC10774117

[175] Yang X (2020) Multitissue multiomics systems biology to dissect complex diseases. Trends Mol Med 26, 718–728. 32439301 10.1016/j.molmed.2020.04.006 PMC7395877

[176] de Graaf AA , Freidig AP , De Roos B , et al. (2009) Nutritional systems biology modeling: from molecular mechanisms to physiology. PLoS Comput Biol 5, e1000554. 19956660 10.1371/journal.pcbi.1000554 PMC2777333

[177] Thiele I , Sahoo S , Heinken A , et al. (2020) Personalized whole-body models integrate metabolism, physiology, and the gut microbiome. Mol Syst Biol 16, e8982. 32463598 10.15252/msb.20198982 PMC7285886

[178] Francis ST & Eldeghaidy S (2015) Imaging methodologies and applications for nutrition research: what can functional MRI offer? Proc Nutr Soc 74, 89–98. 25342449 10.1017/S0029665114001530

[179] Burton-Pimentel KJ , Pimentel G , Hughes M , et al. (2021) Discriminating dietary responses by combining transcriptomics and metabolomics data in nutrition intervention studies. Mol Nutr Food Res 65, e2000647. 33325641 10.1002/mnfr.202000647 PMC8221028

[180] Badimon L , Vilahur G & Padro T (2017) Systems biology approaches to understand the effects of nutrition and promote health. Br J Clin Pharmacol 83, 38–45. 27062443 10.1111/bcp.12965 PMC5338131

[181] Shamanna P , Joshi S , Shah L , et al. (2021) Type 2 diabetes reversal with digital twin technology-enabled precision nutrition and staging of reversal: a retrospective cohort study. Clin Diabetes Endocrinol 7, 21. 34776010 10.1186/s40842-021-00134-7 PMC8591797

[182] FSO (2024) Healthcare Pocket Statistics 2024. Wiesbaden: Federal Statistical Office.

[183] Zheng P , Afshin A , Biryukov S , et al. (2022) The burden of proof studies: assessing the evidence of risk. Nat Med 28, 2038–2044. 36216935 10.1038/s41591-022-01973-2 PMC9556298

[184] Murray CJ , Ezzati M , Lopez AD , et al. (2003) Comparative quantification of health risks conceptual framework and methodological issues. Popul Health Metr 1, 1. 12780936 10.1186/1478-7954-1-1 PMC156894

[185] Gao Q , Praticò G , Scalbert A , et al. (2017) A scheme for a flexible classification of dietary and health biomarkers. Genes Nutr 12, 34. 29255495 10.1186/s12263-017-0587-x PMC5728065

[186] Dragsted LO , Gao Q , Scalbert A , et al. (2018) Validation of biomarkers of food intake-critical assessment of candidate biomarkers. Genes Nutr 13, 14. 29861790 10.1186/s12263-018-0603-9 PMC5975465

[187] Gal R , Monninkhof EM , van Gils CH , et al. (2021) Effects of exercise in breast cancer patients: implications of the trials within cohorts (TwiCs) design in the UMBRELLA fit trial. Breast Cancer Res Treat 190, 89–101. 34427806 10.1007/s10549-021-06363-9 PMC8557193

[188] Krieger JP , Pestoni G , Cabaset S , et al. (2018) Dietary patterns and their sociodemographic and lifestyle determinants in switzerland: results from the national nutrition survey menuCH. Nutrients 11, 62. 30597962 10.3390/nu11010062 PMC6356790

[189] San-Cristobal R , Navas-Carretero S , Celis-Morales C , et al. (2015) Analysis of dietary pattern impact on weight status for personalised nutrition through on-line advice: the Food4Me Spanish cohort. Nutrients 7, 9523–9537. 26593942 10.3390/nu7115482 PMC4663610

[190] Tsiampalis T , Faka A , Psaltopoulou T , et al. (2021) The relationship of the built and food environments with the metabolic syndrome in the Athens metropolitan area: a sex-stratified spatial analysis in the context of the ATTICA epidemiological study. Hormones (Athens) 20, 723–734. 33860926 10.1007/s42000-021-00293-3

